# Breast Tumor Classification Using an Ensemble Machine Learning Method

**DOI:** 10.3390/jimaging6060039

**Published:** 2020-05-29

**Authors:** Adel S. Assiri, Saima Nazir, Sergio A. Velastin

**Affiliations:** 1 College of Business, King Khalid University, Abha 62529, Saudi Arabia; adaseri@kku.edu.sa; 2 Department of Software Engineering, Fatima Jinnah Women University, The Mall Rawalpindi, Punjab 46000, Pakistan; drsaima.nazir@fjwu.edu.pk; 3 Applied Artificial Intelligence Research Group, Department of Computer Science, Universidad Carlos III de Madrid, 28270 Colmenarejo, Spain; 4 School of Electronic Engineering and Computer Science, Queen Mary University of London, London E1 4NS, UK; 5 Zebra Technologies Corporation, London SE1 9LQ, UK

**Keywords:** breast cancer tumor, classification, majority-based voting mechanism, multilayer perceptron learning network, simple logistic regression, stochastic gradient descent learning, wisconsin breast cancer dataset

## Abstract

Breast cancer is the most common cause of death for women worldwide. Thus, the ability of artificial intelligence systems to detect possible breast cancer is very important. In this paper, an ensemble classification mechanism is proposed based on a majority voting mechanism. First, the performance of different state-of-the-art machine learning classification algorithms were evaluated for the Wisconsin Breast Cancer Dataset (WBCD). The three best classifiers were then selected based on their F3 score. F3 score is used to emphasize the importance of false negatives (recall) in breast cancer classification. Then, these three classifiers, simple logistic regression learning, support vector machine learning with stochastic gradient descent optimization and multilayer perceptron network, are used for ensemble classification using a voting mechanism. We also evaluated the performance of hard and soft voting mechanism. For hard voting, majority-based voting mechanism was used and for soft voting we used average of probabilities, product of probabilities, maximum of probabilities and minimum of probabilities-based voting methods. The hard voting (majority-based voting) mechanism shows better performance with 99.42%, as compared to the state-of-the-art algorithm for WBCD.

## 1. Introduction

Breast cancer is one of the leading causes of death among women. According to a 2013 World Health Organization report, “It is estimated that over 508,000 women died worldwide in 2011 due to breast cancer”. Breast cancer can be cured and prevented in the primary stages. However, many women are diagnosed with cancer when it is too late. Breast cancer occurs in the breast cells, fatty tissues or fibrous connective tissues within the breast. Breast cancer tumors tend to gradually worsen and grow faster, which causes death [1]. Although it is more common among women, it can also occur among men. Different factors such as age and family history can also increase the risk of breast cancer. Breast cancer tumors are classified into two classes, benign and malignant [2]. A benign tumor is not dangerous for the human body and rarely causes death in humans. This type of tumor grows in one part of the body and has limited growth. A malignant tumor is more dangerous and can cause death in humans. This type of tumor grows rapidly because of the abnormal growth of cells.

The main types of breast cancer are invasive ductal carcinoma, ductal carcinoma in situ and invasive lobular carcinoma. Ductal carcinoma in situ is the earliest stage of breast cancer and is curable. Invasive ductal carcinoma originates in the milk duct and is the most common breast cancer. Invasive lobular carcinoma can quickly spread to lymph nodes and other areas of the body. It starts in a lobule of the breast.

Approximately one million women are diagnosed with breast cancer every year worldwide. In the early stage, the rate of survival can be high; the five-year survival rate at this stage is 81%. However, only 35% women with late or advanced-stage breast cancer survive for five years. Turkki et al. [3] stated that prognostic assessment using machine learning techniques is possible without a prior knowledge of breast cancer pathology.

This paper proposes a novel ensemble classification method for breast tumor classification using machine learning classification methods. We evaluated the performance of the following classification algorithms: simple logistic Regression learning, SVM learning with stochastic gradient descent optimization and multilayer perceptron network, random decision tree method, random decision forest method, SVM learning with sequential minimal optimization, K-nearest neighbor classifier, and Naïve Bayes classification. The prediction of the three best classification algorithms are then used for ensemble classification. For ensemble classification, we used unweighted voting mechanisms including majority-based voting and four minimum probabilities, maximum probabilities, product of probabilities, and the average of probabilities. The performance of the proposed approach is evaluated on the publicly available Wisconsin Breast Cancer Dataset (WBCD).

The rest of the paper is arranged as follows: Section 2 reviews existing work on breast cancer tumor detection and discusses the importance of the use of appropriate performance measure in medical diagnostics. In Section 3 the proposed methodology is explained in detail, followed by the experimentation and results discussion in Section 4 and Section 5. Section 7 concludes the paper and suggests some future direction of research for breast cancer tumor classification.

## 2. Related Work

Many researchers have emphasized the importance of AI and deep learning in healthcare for the delivery of improved quality and safety of care [4,5,6,7,8,9,10,11,12]. Bejnordi et al. [8] used deep learning algorithm to diagnose breast cancer tumors and compared performance with pathologists’ diagnoses. Results showed that automatic detection using deep learning algorithm outperformed human diagnosis. Khan et al. [13] combined features extracted via GoogleNet, VGGNet and ResNet by using transfer learning.

Haifeng et al. [14] proposed a novel method for breast cancer prediction using data mining techniques. They formulated an effective way to predict breast cancer based on patients’ clinical records. They evaluated the method on two popular publicly available datasets: the Wisconsin Breast Cancer Database (WBCD) and the Wisconsin Diagnostic Breast Cancer (WDBC) dataset. They evaluated the performance of support vector machines (SVMs), eight learning models, artificial neural networks (ANNs), Naïve Bayes classification and AdaBoost Tree. They proposed a combined model based on principal component analysis and other data mining models for feature reduction and suggested that other models such as k-mean could be used for feature space reduction.

In contrast, Quang et al. [15] used both supervised and unsupervised classification models for breast tumor classification. They proposed a combination of scaling and principal component analysis for feature selection. They proved that an ensemble voting approach is the best prediction model. After feature selection, various classification models were tested and trained on the data. Among all the models used for the prediction, only four models: ensemble-voting classifier, logistic regression (LR), SVM and AdaBoost showed better performance, with accuracies of around 90% based on the models’ results for precision and recall, Area Under the Receiver Operating Characteristics (AUC-ROC), F1 measure and computational time.

Ahmed et al. [16] used decision trees (DTs), ANNs and SVMs. The implementation of different algorithms indicated that SVMs [17] outperform other classifiers on the WBCD. DT, ANN and SVMs showed 93.6%, 94.7% and 95.7% accuracy, respectively. They used 10-fold cross validation for evaluation.

Mandal et al. [18] used LR, Naïve Bayes (NB) and DTs. They also analyzed the time complexity of each classifier. LR outperforms other classifiers with the highest accuracy. However, Borges et al. [19] evaluated the performance of Bayesian networks and DT and found that Bayesian networks performed better, with 97.80% accuracy.

Chaurasia et al. [20] used Bayesian theorem, DT, radial basis function network and J48. They evaluated the results on the WBCD dataset and applied pre-processing, data selection and data transformation to form a prediction model. They found that Naïve Bayes outperformed other models with a classification accuracy of 97.36%. Radial Basis Function (RBF) Network and J48 showed classification accuracies of 96.77% and 93.41%, respectively.

Kumar et al. [21] predicted breast cancer using twelve classification algorithms: AdaBoost, J-Rip, LR, lazy learner, decision table, IBK, J48, lazy K-star, multiclass classifier, multilayer perceptron, random forest, Naïve Bayes and random tree. They stated that other than Naïve Bayes classification, the algorithms performed with accuracy greater than 94% and that lazy and tree classifications outperformed other classification algorithms, with 99% accuracy.

Lee et al. [22] stated that although ensemble learning enables the improvement of performance of a base learner, it decreases the bias or variance. Abdar et al. [23] proposed a new ensemble classification algorithm, CWV-BANNSVM, by combining Boosting Artificial Neural Network (BANN) along with two SVMs to improve the performance for WBCD. In contrast to traditional ensemble learning, Alam et al. [24] proposed a novel dynamic ensemble learning algorithm to automatically determine the number of neural networks and their architecture. Different training sets are used for each neural network hence guaranteeing better learning from the whole training data samples. The proposed DEL is trained several times to find the correct values of learning rate parameter and the correlation strength parameter by using an incremental training approach. Osman et al. [25] stated that improvement is possible when using the ensemble boosting method. The method was integrated with a Radial Basis Function neural network algorithm and performance was increased to an accuracy of 98.4% for the WBCD dataset.

Most of the reported literature evaluates the performance of the proposed method based on ‘accuracy’. Accuracy is higher when the occurrence of true positives (TPs) and true negatives (TNs) is high compared to the false positives (FPs) and false negatives (FNs). Other than accuracy, precision and recall are also important for performance reporting. However, for medical diagnostic the performance of artificial intelligence systems should give more importance to false negatives (recall) than false positives (precision), because missing a condition can have serious consequences for patients. Therefore, it is important to evaluate the performance based on f-measures (e.g., F2, F3) that weigh recall more than precision.

## 3. Methodology

**Figure 1:**
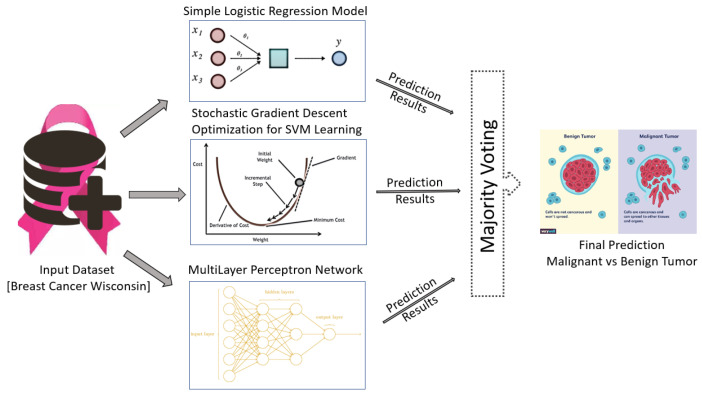
An ensemble method based on majority-based voting mechanism for breast cancer tumor classification using different machine learning models.

In this paper, a novel algorithm is proposed for breast tumor classification using a voting mechanism. The publicly-available WBCD is used for evaluation and comparisons are made with existing state-of-the-art methods. As shown in Figure 1, the input dataset is supplied to the different classification learning models to detect and differentiate malignant and benign tumors.

The performance of eight different state-of-the-art machine learning classification algorithms were tested for tumor classification: simple logistic regression model, SVM learning with stochastic gradient descent optimization, multilayer perceptron network, random decision tree method, random decision forest method, SVM learning with sequential minimal optimization, k-nearest neighbor classification and Naïve Bayes classification algorithm.

These classifiers are evaluated and three classification algorithms are selected based on the best performance calculated using an f-measure. These selected classifiers are used to propose a new ensemble classification method based on a voting mechanism. In this paper, two different voting mechanism were evaluated: hard voting (majority-based voting) and soft voting. Soft voting consist of either average of probabilities voting, product of probabilities, minimum of probabilities or maximum of probabilities voting.

The classification results of the three best classifiers are supplied to the ensemble classification method, which provides a final classification result. Details of the classification models and voting mechanism used are given in the following sub-sections.

### 3.1. Classification Methods

#### 3.1.1. Simple Logistic Regression Model

The LR classification model [26] is a popular choice for modeling binary classifications. For this model, the conditional probability of one of the two output classes is assumed to be equal to a linear combination of the input features [27]. The logistic equation used for this classification model is:

(1) $$Z_{i}=\;ln\left(\frac{P_{i}}{1-\;P_{i}}\right)$$

where *P* is the probability of the occurrence of event *i*.

#### 3.1.2. SVM Learning with Stochastic Gradient Descent (SGD) Optimization

We used SGD for support vector machine classification algorithm with hinge loss function. Gradient descent is one of the most popular optimization algorithms used in deep learning and machine learning algorithms. Stochastic gradient descent [28] selects random samples from a dataset instead of selecting the batch data as in batch gradient descent. Stochastic gradient descent optimization randomly shuffles samples in the training data set. It computes gradient and performs weight updates for each selected training sample $x^{\left(i\right)}$ and the label $output^{\left(i\right)}$ until a minimum cost $\left(J_{min}\left(w\right)\right)$ is reached.

(2) $$\begin{array}{c} \mathrm{Randomly}\;\mathrm{shuffle}\;\mathrm{samples}\;\mathrm{in}\;\mathrm{the}\;\mathrm{training}\;\mathrm{set}\\ \mathrm{Until}\;\mathrm{approx}.\;\mathrm{cost}\;\mathrm{minimum}\;(J_{min}\left(w\right))\;\mathrm{is}\;\mathrm{reached:}:\\ \mathrm{For}\;\mathrm{each}\;\mathrm{training}\;\mathrm{sample}\;i:\\ \mathrm{Compute}\;\mathrm{gradients}\;\mathrm{and}\;\mathrm{perform}\;\mathrm{weight}\;\mathrm{updates}\;\\ \mathrm{For}\;\mathrm{each}\;\mathrm{weight}\;j\\ w_{j}=w+\Delta w_{j},where:\Delta w_{j}=\eta \left({\mathrm{target}}^{\left(i\right)}-{\mathrm{output}}^{\left(i\right)}\right)x_{j}^{\left(i\right)},\;\eta \;\mathrm{is}\;\mathrm{learning}\;\mathrm{rate} \end{array}$$

#### 3.1.3. Multilayer Perceptron Network

A multilayer perceptron (MLP) [29] is an artificial neural network that contains many perceptrons. It is traditionally structured into three groups of layers, the input layer, the output layer and the (hidden) layers between these two. A deep network consists of many hidden layers. These hidden layers hold the key to multilayer perceptron computation. MLPs are often applied on supervised learning algorithms and learn the model based on the correlation between input and output variables.

#### 3.1.4. Random Decision Tree

The random decision tree is one of the popular supervised machine learning algorithms used for the graphical representation of all the possible solutions [30]. The decisions are based on some conditions and are easy to interpret. It identifies and chooses the significant attributes that are helpful in classification. It selects only those attributes that return the highest information gain (*IG*). *IG* is defined as:

(3) IG=E(ParentNode)−AverageE(ChildNodes)

where Entropy (*E*) is defined as:

(4) $$E\;=\;\sum_{i}-Prob_{i}\;\left(\log_{2}Prob_{i}\right)$$

and $Prob_{i}$ is the probability of class *i*.

#### 3.1.5. Random Decision Forest

Random decision forest is similar to the bootstrapping algorithm with decision tree (CART) model. Random decision forest tries to build *k* different decision trees by picking a random subset *S* of training samples [31]. It generates the full Iterative Dichotomiser 3 (ID3) [32] trees with no pruning. It makes a final prediction based on the mean of each prediction. Random decision trees can interpret and handle irrelevant attributes in a simple manner. They are compact and can handle missing data.

#### 3.1.6. SVM Learning with Sequential Minimal Optimization (SMO)

SMO [33] is the optimization method for Support Vector Machine (SVM) [34] training problem. SMO is more efficient than the quadratic programming solvers and uses heuristics to divide the whole training problem into minor problems so that these smaller problems can be solved analytically. Usually, it reduces training time by a wide margin. SMO uses “John Platt’s sequential minimal optimization algorithm” [35] for training an SVM [36].

#### 3.1.7. K-Nearest Neighbor Classification

KNN [37] stores all the training data and classifies the query data based on a similarity measure. In KNNs, *k* is used to refer to the number of nearest neighbors that are to be included in the voting processes. KNNs use feature similarity. To get better performance, KNN parameter tuning is done by choosing an appropriate value of *k*. The similarity between two points is calculated using, for example, the Euclidean distance.

#### 3.1.8. Naïve Bayes Classification

The Naïve Bayes classification algorithm [38] is normally famous for its simplicity as well as effectiveness. The Naïve Bayes classification model is fast to build and it makes quick predictions. Naïve Bayes is a probabilistic classifier and it learns the probabilities of features based on the target class. It assumes that the occurrence of a particular attribute is independent of the occurrence of the other attributes. Even if it depends on the other attributes, Naïve Bayes can show better performance as it does not require accurate probabilities estimates provided that the highest probability is allocated to the correct class. It is based on the Bayes theorem which states that:

(5) $$P\left(A|B\right)\;=\;\frac{P(B|A)\;P(A)}{P(B)}$$

where *P(A|B)* and *P(B|A)* are the conditional probabilities of occurrence of an event *A* given that event *B* is true and vice versa. *A, P(A), P(A|B)* and *P(B|A)* are called proposition, prior probability, posterior probability and likelihood, respectively.

### 3.2. Voting Mechanism

#### 3.2.1. Majority-Based Voting Mechanism (Hard Voting)

Majority-based voting [39] is widely used in ensemble classification. It is also known as plurality voting. In the approach proposed here, after applying the three above-mentioned classification algorithms, a majority-based voting mechanism is used to improve the classification results [40]. Each of these model classification results is computed for each test instance and the final output is predicted based on the majority results. In majority voting, the class label *y* is predicted via majority (plurality) voting of each classifier *C*:

(6) $$y\;=\;mode\;\left\{\;C_{1}\left(x\right),\;C_{2}\left(x\right),\;..,\;C_{n}\left(x\right)\right\}$$

#### 3.2.2. Soft Voting

In soft voting, the final output is predicted based on the predicted probabilities *p* for the classifiers [41]. In each case below, the probability of class labels assigned by the classifier *C* to input *x* is defined as:

(7) $$\mathrm{Average}\;\mathrm{of}\;\mathrm{Probabilities}\;\mathrm{Voting}:\;y=\;AVERAGE\;\left\{\;C_{1}\left(x\right),\;C_{2}\left(x\right),\;.....,{\;C}_{L}\left(x\right)\;\right\}$$

(8) $$\mathrm{Product}\;\mathrm{of}\;\mathrm{Probabilities}\;\mathrm{Voting}:\;y=\;PROD\;\left\{\;C_{1}\left(x\right),\;C_{2}\left(x\right),\;.....,{\;C}_{L}\left(x\right)\;\right\}$$

(9) $$\mathrm{Minimum}\;\mathrm{of}\;\mathrm{Probabilities}\;\mathrm{Voting}:\;y=\;MIN\;\left\{\;C_{1}\left(x\right),\;C_{2}\left(x\right),\;.....,{\;C}_{L}\left(x\right)\;\right\}$$

(10) $$\mathrm{Maximum}\;\mathrm{of}\;\mathrm{Probabilities}\;\mathrm{Voting}:\;y=\;MAX\;\left\{\;C_{1}\left(x\right),\;C_{2}\left(x\right),\;.....,{\;C}_{L}\left(x\right)\;\right\}$$

## 4. Results

The publicly available WBCD dataset was analyzed (it can be downloaded from the UCI repository [42]). This dataset contained data taken from microscopic examination of breast masses. A digital scan of fine-needle aspirates was used to compute features. Fine-needle aspirate is one of the best methods to evaluate the presence of malignant tumors.

**Figure 2:**
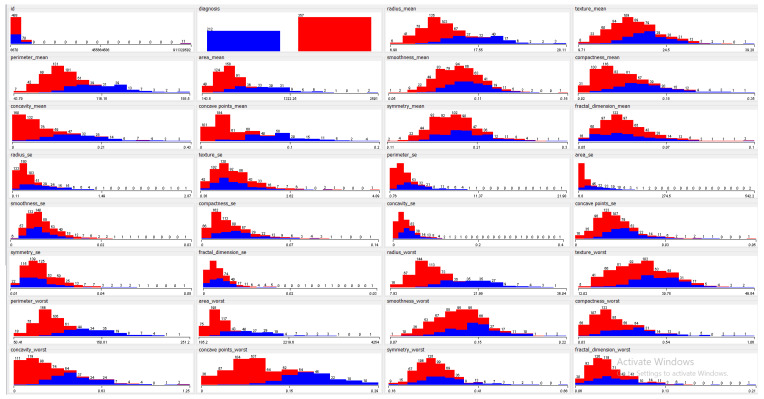
Feature visualization result for WBCD (x-axis represents the attribute value and y-axis represents the frequency of each value.

This dataset was comprised of 569 instances. Each instance contained 32 features that were extracted from images of nuclei. These include the nucleus texture, radius, perimeter, smoothness, area, compactness, concave points, concavity, fractal dimension and symmetry. The remaining features were computed by taking the mean, standard error and worst or largest mean of the above-mentioned features. Figure 2 shows the univariate attribute distribution of the above-mentioned features where the x-axis represents the attribute value and the y-axis represents the frequency of each value for both classes. The first and second features shown in Figure 2 are ID and diagnosis class (malignant/benign). Results shown in red and blue represent benign and malignant classes, respectively. A 70%–30% training-testing split was used for evaluation, as commonly reported in the literature.

### Performance Evaluation Measures

Given the true positive (*TP*), false positive (*FP*), true negative (*TN*) and false negative (*FN*) counts, the following performance evaluation measures were calculated:

(11) $$Accuracy\;=\;\frac{TP+\;TN}{TP\;+\;FP\;+\;TN\;+\;FN}$$

(12) $$Precision\;=\;\frac{TP}{TP\;+\;FP\;}$$

(13) $$Recall=\;\frac{TP}{TP+\;FN}$$

(14) $$F_{\beta }=\;\left(1+\beta^{2}\right)\;\frac{Precision\;*\;Recall}{{(\beta }^{2}\;*\;Precision)+\;Recall}\;$$

where $\beta^{2}$ is a positive real number. For F1, F2, F3 score calculation, β is set to 1, 2 and 3 respectively.

In medical diagnostics research, precision and recall are the main metrics used for reporting performance. F-measure is also important in reporting performance in medical diagnostics, because it combines precision and recall into a single figure which is simpler to use for comparisons. In the medical community, a false negative is normally more devastating than a false positive therefore, recall is considered to be a more important measurement. An FP (precision) might not be as important as an FN (recall) because FPs can be discarded by clinicians but a missed condition could be very serious for a patient. In the light of that, a modified form of F1 that weighs recall more heavily than precision is a better metric. Therefore in this work F2 and F3 were used.

## 5. Discussion

The performances of eight different classification algorithms are evaluated on the WBCD testing dataset. Table 1 shows the computational time for these classification algorithms. Multilayer perceptron network takes longer than the other algorithms. For simple logistic regression, the L2 regularization method was used with the conjugate gradient (CG) method for optimization. SGD used optimal learning rate with alpha = 0.0001 and L2 regularization method for SVM. The hidden layer size for MLP was set to 100, with ‘relu’ activation function and stochastic gradient-based optimizer for weight optimization with learning rate set to 0.002. Random decision tree used Gini impurity to split the tree. The nodes were expanded until all leaves are pure or until all leaves contained less than two. An SMO optimization method normalized the training data and polynomial kernel for classification using SVM. For KNN, k = 4 nearest neighbors were searched using an Euclidean distance measure, and each neighbor was weighted equally for classification purposes. For Naïve Bayes classification, numeric estimator precision values were chosen based on the analysis of the training data.

**Figure 3:**
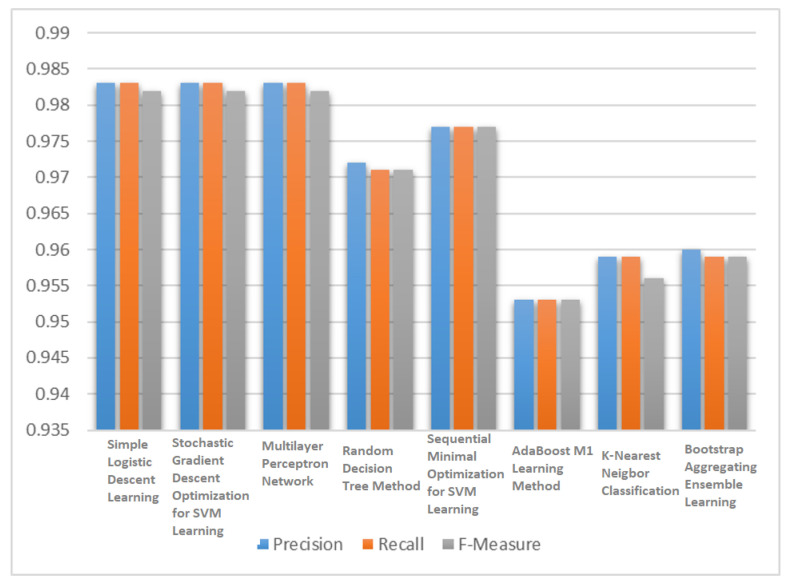
F3 score of the classification algorithms for WBCD.

Table 2 shows the results for testing data on WBCD. The SLR model chose the attributes that resulted in the lowest possible squared error. SGD learning removed all the missing values and converts nominal attributes into binary ones. All attributes were also normalized. MLP learning used a back propagation algorithm for learning. All the nodes in MLP network were sigmoid. At each node, random decision tree considered randomly chosen k attributes without performing pruning. The SMO method made use of John Platt’s optimization algorithm for training SVM. KNN selected an appropriate *k* based on cross validation and performs distance weighting for learning. Figure 3 compared the performance of the all eight classifiers in terms of F3 score. The simple logistic regression (SLR) learning model, SVM learning with stochastic gradient descent (SGD) optimization and multilayer perceptron network (MLP) showed better performance in terms of F3 score than the other five classification algorithms.

After analyzing these results, the three best classifiers were selected based on F3 score i.e., simple logistic learning, SVM learning with stochastic gradient descent optimization and multilayer perceptron network and passed their classification results to different voting mechanisms to predict a final output. For ensemble method classification, different unweighted voting schemes were used i.e., minimum probabilities, maximum probabilities, majority voting, product of probabilities and the average of probabilities.

Table 3 shows the results of the voting mechanisms for the WBCD. The results showed that majority-based voting performed better than the other voting mechanisms. Usually it is assumed that soft voting performs better than hard voting as it considers more information by using individual classifier’s uncertainty in the final prediction. However, these experiments show that for WBCD, the selected classification algorithms and majority-based voting (hard voting) performs better than soft voting mechanisms i.e., average of probabilities voting, product of probabilities, maximum of probabilities and minimum of probabilities. This might be explained because of the low confidence (low probability) of the selected classification algorithms. All these results reported are for 70:30 training testing split. For 10, 5 and 2 fold cross validation, the proposed algorithm achieved accuracies of 98.77%, 98.43%, and 99.23% respectively.

## 6. Comparison with Existing Work

Table 4 shows a comparison between the proposed majority-based voting mechanism with existing work on breast cancer prediction using the WBCD. Nahato et al. [43] used a back propagation neural network. Chen et al. [44] Kumari et al. [45] and Dumitru et al. [46] used conventional prediction algorithms like support vector machines, K-nearest neighbor and Naïve Bayes, respectively. Liu et al. [47] proposed a novel evolutionary neural network approach and Nguyen et al. [15] used feature scaling and principle component analysis prior to model training. Osman et al. [25] enhance the performance of radial based function neural network model (RFBNN) by integrating the neural network with ensemble features using a boosting method. As far as we can ascertain, the proposed approach performs better than any in the literature to date.

## 7. Conclusions

Breast cancer offers a unique context for medical diagnosis by also considering the patients’ condition, and their response to treatment. Machine learning has shown considerable improvement for breast cancer diagnosis. However, challenges remain for accurate diagnosis and monitoring of breast tumors despite improved technologies. There is a need to integrate the biological, social and demographic stream of information to improve the predictive models. We have proposed here an ensemble classification by evaluating the performance of simple logistic regression learning, SVM learning with stochastic gradient descent optimization and multilayer perceptron network, random decision tree, random decision forest, support vector machine learning with sequential minimal optimization, K-nearest neighbor classifier, and Naïve Bayes classification algorithms. We have reported the performance of these eight classifiers using different performance measures i.e., accuracy, precision, recall, F1 score, F2 score, and F3 score. Then, we selected the three classifiers with the best F3 score and proposed an ensemble method using a voting mechanism. We used different unweighted voting mechanisms including majority-based voting, average of probabilities, product of probabilities, minimum of probabilities and maximum of probabilities. The majority-based voting mechanism shows better F3 score than the other voting mechanism with results that outperform the state-of-the-art, as far as we can establish.

For future research, we plan to evaluate different feature selection algorithms that can help us determine the smallest subset of features that can assist in accurate classification of breast cancer as either benign or malignant. Instead of an unweighted voting mechanism, researchers can evaluate the performance of different weighted voting mechanisms including simple weighted voting, rescaled weighted voting, best-worst weighted voting and quadratic best-worst weighted voting. It would be useful to use other datasets for breast cancer classification to evaluate and improve the performance of proposed algorithm. The findings of the present study can be a good starting point for breast tumor classification using the image dataset too.

## Figures and Tables

**Table 1:** Computational times for the classification algorithms.

Classification Algorithm	Computational Time (s)
Simple logistic regression model	0.34
SVM learning with SGD optimization	0.13
Multilayer perceptron network	3.10
Random decision tree method	0.01
Random decision forest method	0.06
SVM learning with SMO	0.30
K-nearest neighbor classification	0.01
Naïve Bayes classification	0.08

**Table 2:** Performance of the machine learning algorithms on WBCD.

Classification Algorithms	Accuracy	Precision	Recall	F1 Score	F2 Score	F3 Score
Simple Logistic Regression Learning	98.25%	0.9830	0.9820	0.9825	0.9822	0.9821
SVM learning with SGD optimization	97.88%	0.9791	0.9789	0.9710	0.9710	0.9710
Multilayer Perceptron Network	97.66%	0.9770	0.9770	0.9770	0.9770	0.9770
Random Decision Tree Method	91.81%	0.9200	0.9180	0.9190	0.9184	0.9182
Random Decision Forest Method	96.49%	0.9650	0.9650	0.9650	0.9650	0.9650
SVM learning with SMO	97.08%	0.9710	0.9710	0.9710	0.9710	0.9710
K-Nearest Neighbor Classification	97.08%	0.9710	0.9710	0.9710	0.9710	0.9710
Naïve Bayes Classification	91.81%	0.9190	0.9180	0.9185	0.9182	0.9181

**Table 3:** Evaluation results of the proposed voting mechanisms for the WBCD.

Voting Mechanism	Accuracy	Precision	Recall	F1 Score	F2 Score	F3 Score
Majority-based	**99.42%**	**0.9940**	**0.9940**	**0.994**	**0.9940**	**0.9940**
Average of probabilities	98.83%	0.989	0.988	0.9885	0.9882	0.9881
Product of probabilities	98.12%	0.9850	0.9850	0.9850	0.9850	0.9850
Minimum of probabilities	98.46%	0.986	0.981	0.9835	0.9820	0.9815
Maximum of probabilities	99.41%	0.9840	0.9840	0.9840	0.9840	0.9840

**Table 4:** Comparison with the existing work for breast cancer prediction using WBCD.

1l Work	Proposed Method	Accuracy
Ours	Majority-based voting mechanism	**99.42% (70:30), 98.77% (10-CV)**
Nahato et al. [43]	Backpropagation neural network	98.60% (80:20)
Liu et al. [47]	An evolutionary artificial neural network	97.38% (60:40)
Chen et al. [44]	A support vector machine classifier with rough set-based feature selection	89.20% (70:30)
Kumari et al. [45]	K-Nearest neighbor classification algorithm	99.28% (10-CV)
Dumitru et al. [46]	Naïve bayesian classification	74.24% (-)
Shaikh et al. [48]	Dimensionality reduction and support vector machine	97.91% (-)
Nguyen et al. [15]	Feature selection and ensemble voting	98.00% (10-CV)
Alickovic et al. [49]	Normalized multi layer perceptron neural network	99.27% (-)
Osman et al. [25]	Ensemble learning using Radial Based Function Neural Network models (RBFNN)	97.00% (10-CV)
Kaushik et al. [50]	Ensemble learning via MLP, RF and RT	93.50% (10-CV)
